# Supplementary material for: Analysing high-throughput sequencing data in Python with HTSeq 2.0
Source: Bioinformatics. 2022 Mar 21;38(10):2943–5. doi: 10.1093/bioinformatics/btac166 (PMC9113351; doi:10.1093/bioinformatics/btac166)
Supplement: btac166_Supplementary_Data [file btac166_supplementary_data.pdf]

# Supplementary Materials for “Analysing high-throughput sequencing data in Python with HTSeq 2.0”

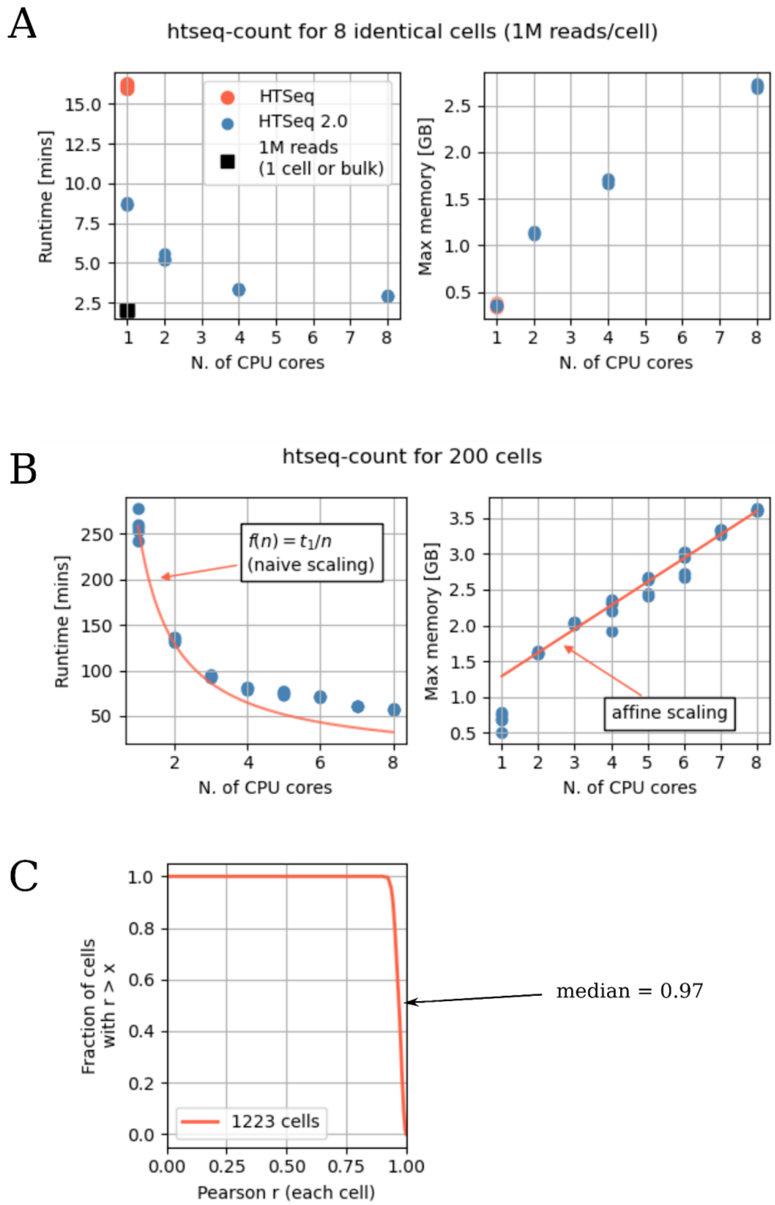


**Supplementary Figure 1: Runtime and memory scaling for htseq-count in HTSeq 2.0 and comparison with cellranger.** (A) Runtime and maximal volatile memory (RAM) for counting features from 8 identical human cells, each a separate BAM file with 1M reads. While memory usage is unchanged from the previous version of HTSeq, runtime is significantly shortened even for a single CPU because the GTF file is parsed only once per run instead of once per cell. Runtime is further reduced by parallel computing, with an apparent tradeoff in maximum memory requirements. Typical time for 1 million bulk

reads plus parsing the GTF file is around 2 minutes, while processing 1 million reads takes around 57-60 seconds. (B) As in (A) but for 200 typical single cells from (Domingo-Gonzalez et al. 2020), which used a modified Smart-seq-2 protocol (Picelli et al. 2013). Runtime scales almost naively or optimally, while a few instructions are executed exactly once (e.g. output to file, merging the count tables, parsing the GTF file) and therefore have incompressible runtime. Memory scaling follows an affine curve, as expected by a central orchestration cost plus the memory allocated to each CPU process. (C) Cumulative distribution of Pearson correlations in gene counts between cellranger count (10X Genomics) and htseq-count-barcodes from HTSeq 2.0, with each data point representing a high-quality cell. All HTSeq benchmarks were run on an Apple Macbook Pro laptop with Apple M1 chip, 8 CPU cores, and 16 GB of RAM. Read mapping and counting using cellranger (v 6.1.2) for benchmark C was run on a workstation with 40 Intel Xeon Platinum 8160T CPUs (80 vCPUs) and 512GB of RAM. Data for panel C available at https://www.10xgenomics.com/resources/datasets/1-k-pbm-cs-from-a-healthy-donor-v-3-chemistry-3-standard-3-0-0. Code for the benchmarks available at https://github.com/ghar1821/htseq_benchmark.

## Supplementary References

Domingo-Gonzalez, Racquel, Fabio Zanini, Xibing Che, Min Liu, Robert C. Jones, Michael A. Swift, Stephen R. Quake, David N. Cornfield, and Cristina M. Alvira. 2020. “Diverse Homeostatic and Immunomodulatory Roles of Immune Cells in the Developing Mouse Lung at Single Cell Resolution.” *eLife* 9 (June). https://doi.org/10.7554/eLife.56890.

Picelli, Simone, Åsa K. Björklund, Omid R. Faridani, Sven Sagasser, Gösta Winberg, and Rickard Sandberg. 2013. “Smart-seq2 for Sensitive Full-Length Transcriptome Profiling in Single Cells.” *Nature Methods* 10 (11): 1096–98.
